# Comparing sugammadex to neostigmine and their effects on delirium and postoperative cognitive function: A systematic review

**DOI:** 10.1177/17504589251379195

**Published:** 2025-11-03

**Authors:** Andrew Slowgrove, Celeste Ng, Alexandra Tebbett

**Affiliations:** Warwick Medical School, University of Warwick, Coventry, UK

**Keywords:** Anaesthetics, Management and care, Anaesthetics, Pharmacology, Post-anaesthetic care, Sugammadex, Neostigmine, Delirium, Postoperative cognitive function, Vecuronium, Rocuronium

## Abstract

**Background:**

Sugammadex is a relatively new therapeutic agent that reverses neuromuscular blockade. Compared to neostigmine, it is hypothesised that sugammadex could have a beneficial effect on postoperative cognitive function, benefitting postoperative recovery.

**Objective:**

To compare the effects of both sugammadex and neostigmine on delirium and postoperative cognitive dysfunction.

**Methods:**

A systematic search of all relevant randomised controlled trials and observational cohort studies was performed in October 2024, utilising the following inclusion criteria: incidence of delirium and postoperative cognitive dysfunction following administration of neostigmine or sugammadex, adult patients, patients given rocuronium or vecuronium, English language studies, papers since the introduction of sugammadex (2008). CASP and Cochrane risk-of-bias tools were utilised for study appraisal, with a narrative synthesis of the results.

**Results:**

Five studies, reporting interventions in 49,910 patients, met the review criteria and were included. Of these, four showed no difference in cognitive function between sugammadex and neostigmine. One cohort study of 21 patients showed favourable outcomes postoperatively in the sugammadex group.

**Conclusion:**

This systematic review suggests the possibility of a very limited neurological protective role of sugammadex compared to neostigmine, but no clinical significance was reported. Only a limited number of studies were available, suggesting the need for further research.

## Introduction

Delirium is an acute condition associated with altered and fluctuating levels of consciousness, attention and cognition (Brodier & Cibelli 2021). It can be categorised into hyperactive, hypoactive or mixed-type, with hypoactive presentations being the most common yet often missed variation. Delirium has been shown to affect 25%–60% of patients aged over 65 years in the postoperative period (Schenning & Deiner 2015). Termed postoperative delirium (POD), it usually presents between 1 and 5 days after surgery (Needham et al 2017, Schenning & Deiner 2015, Zhaoshen et al 2020,) and is associated with increases in both morbidity and mortality, including significant functional decline (Schenning & Deiner 2015).

Comparatively, postoperative cognitive dysfunction (POCD) generally occurs 1 week to 1 year after surgery (Needham et al 2017). POCD is more challenging to define, with no consensus on definition or standardised diagnostic tests for the condition. It is characterised by a decline in cognition, involving memory, attention and executive function, and affects 10%–65% of patients postoperatively (Brodier & Cibelli 2021, Zhao et al 2024).

POCD negatively impacts physical function, length of hospital stay, and mortality outcomes (Suraarunsumrit et al 2024). Quality of life can be affected due to the detrimental effects of POCD on mental health and the independence of the patient (Berger et al 2015, Brodier & Cibelli 2021). Both postoperative neurocognitive disorders also impact public health, as they are associated with an increase in health and social care costs following surgery (Boone et al 2020).

Postoperative cognitive function is influenced by many factors including the age of the patient, degree of prior cognitive impairment, associated medical conditions and polypharmacy, the type of surgery and the type of anaesthetic (Batistaki et al 2017, Brodier & Cibelli 2021, Davis et al 2014, Zhao et al 2024). Cardiac surgery has been associated with higher incidences of POCD (Vu & Smith 2022).

While the cause is multifactorial, there is an association between postoperative cognitive function and the alterations in acetylcholine seen in response to surgery (Yang et al 2023). Acetylcholine is a neurotransmitter important for memory, attention and sensory information and can cause cognitive decline if deficient, or if the cholinergic receptors are excessively blocked. Conversely, overstimulation of cholinergic receptors can also result in an altered mental status. Anticholinergics, which decrease cholinergic neurotransmission, can therefore precipitate delirium (Herrmann et al 2022). Acetylcholinesterase, an enzyme found in post-synaptic junctions, rapidly hydrolyses acetylcholine, terminating the neuronal transmission and allowing new transmissions to occur. Low or altered acetylcholinesterase activity can also lead to cognitive dysfunction in patients (Caeiro et al 2021).

Neostigmine is an acetylcholinesterase inhibitor used to reverse a neuromuscular block. By inhibiting acetylcholinesterase, neostigmine causes an increase in acetylcholine at the neuromuscular junction. The increase in acetylcholine displaces the competitive inhibition of the non-depolarising neuromuscular blocking drugs allowing acetylcholine to bind so muscles can contract. Neostigmine is postulated to improve cognitive function postoperatively by reducing the inflammatory response to surgery (Zhu et al 2020). However, neostigmine does not always fully reverse the neuromuscular block, leaving residual amounts of non-depolarising agents which can have detrimental effects on cognition via processes such as hypoxia, hypercapnia and other pulmonary complications (Blum et al 2024, Luo et al 2018, Oh et al 2016).

Sugammadex is a modified gamma cyclodextrin designed to completely encapsulate rocuronium and vecuronium. It is more effective as a reversal agent than neostigmine and has minimal cholinergic side effects (Hristovska et al 2017). This newer drug has been proven to allow faster recovery times and superior reversal of deep neuromuscular blocks (Moss et al 2022). It is hypothesised that sugammadex may therefore reduce the incidence of postoperative delirium and POCD when compared to neostigmine use.

A Cochrane review published in 2017 was unable to compare the effects of the two medications on cognitive function, due to the data format being ineligible for meta-analysis (Hristovska et al 2017). No narrative synthesis of this outcome was performed. This article aims to systematically review all relevant studies comparing sugammadex and neostigmine on postoperative cognitive function in adult patients (over 18 years) when administered to reverse vecuronium or rocuronium during general anaesthesia (GA).

## Method

This systematic review follows the PRISMA guidelines (Page et al 2021). The protocol was registered with Prospero (registration number: CRD42024596906) on 8 October 2024. The only adjustment to the original protocol published on Prospero was a change to the inclusion criteria of patient age (changed from > 65 years old to > 18 years old) due to both a lack in consensus on the definition of older age, and papers not publishing age-specific data breakdowns. There was no need for ethical clearance.

### Search strategy

The eligibility criteria were created using the aims of the systematic review, utilising the population, intervention, comparison and outcome (PICO) model.

### Inclusion criteria

(1) Population:
  a. Adult patients (aged 18 years or older)
  b. Patients given rocuronium or vecuronium as a muscle relaxant
(2) Intervention:
  a. Patients given sugammadex as a reversal agent
(3) Comparison:
  a. Patients given neostigmine as a reversal agent
(4) Outcome:
  a. The incidence of delirium and/or postoperative cognitive decline
(5) Additional inclusion criteria:a
  a. English language studies
  b. Studies published since the introduction of sugammadex in the United Kingdom (2008)

### Exclusion criteria

(1) Patients under the age of 18
(2) Papers written in a foreign language with inaccessible translations. In these cases, an attempt to contact the authors will be made
(3) Use of alternative muscle relaxants, for example, cisatracurium
(4) Case studies, descriptive reviews, letters or opinion articles.
(5) Any other non-primary studies, for example, systemic reviews, guidelines

**Figure 1:**
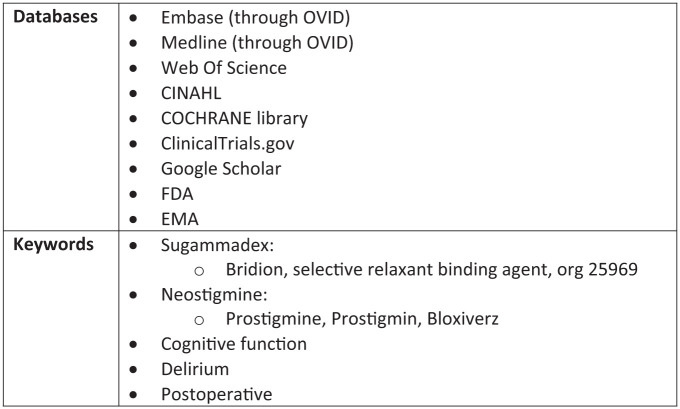
Databases and keywords identified for the search

After consultation with an expert librarian, the databases and search terms were identified as shown is Figure 1. The full search strategy has been included in Appendix 1.

The only restriction placed was references published from 2008 onwards.

### Selection process

The initial references were uploaded to the EndNote (EndNote Team 2013) software and put through the PRISMA (Page et al 2021) flow diagram before being screened independently by two reviewers. The screening was done initially via the reference’s abstract, with the full report used if any uncertainty or conflict arose. Rayyan (Ouzzani et al 2016) was used to facilitate fully blind screening, to reduce the risk of bias. Two reviewers screened all references before unblinding, and any conflicts were discussed and resolved with the third reviewer. The screening was performed using the pre-defined inclusion and exclusion criteria.

### Data collection and management

The data was then extracted into Microsoft Excel (Microsoft Corporation, 2018), with a second reviewer independently verifying that the data had been appropriately gathered. Once the overall study, patient and anaesthetic characteristics of each report were extracted, outcome specific data was collected. This data included: the methods of assessing for postoperative cognitive dysfunction, reported narrative results, statistical results, author conclusions and author declarations. The data was then analysed by two reviewers independently to produce a narrative synthesis, with all authors agreeing on results drawn and conclusions made.

### Risk of bias assessment

The papers were assessed for bias and usefulness by two independent reviewers using the Cochrane risk-of-bias tools Rob-2 (Sterne et al 2019) and ROBINS-I (Sterne et al 2016), and the Critical Appraisal Skills Programme (CASP) checklist for randomised control trials (CASP 2023a) and cohort studies (CASP 2023b). A third reviewer was used to settle any conflicts.

## Results

### Literature search

**Figure 2:**
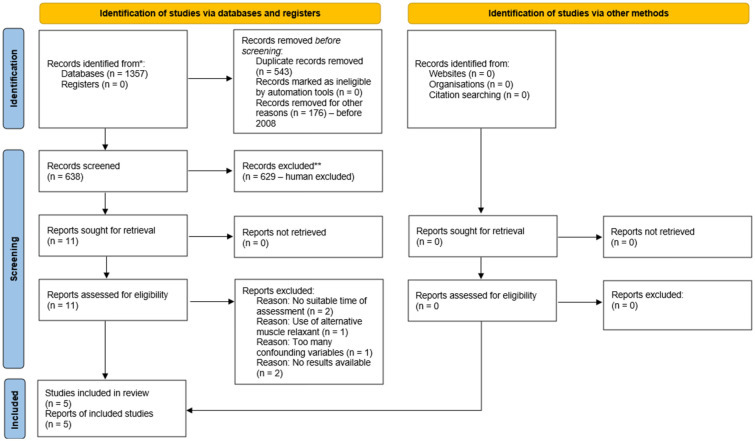
PRISMA flowchart with breakdown for this review

The search was performed on 18 October 2024, with 1357 records initially identified. These records were screened following the PRISMA (Page et al 2021) flowchart (Figure 2) using EndNote (EndNote Team 2013) software. A total of 543 duplicates were removed, and a further 176 records were removed as they were published before 2008, an exclusion criterion as sugammadex was introduced in 2008. After this initial removal, 638 records were transferred to Rayyan (Ouzzani et al 2016) software for blinded abstract-based screening by two independent reviewers. Out of which, 627 records were excluded for not matching the set eligibility criteria. Of the remaining 11 references, one was removed due to unsuitable timings of the cognitive assessments: Claroni et al (2019) performed the assessment within 4 h after the surgery. A study by Kim et al (2019) was removed as they did not publish specific values, only a graph. There was no reply when the authors were contacted for a breakdown of the data in their graph. A paper by Amorim et al (2014) was removed for using an unspecified muscle relaxant: ten out of 48 patients received ‘other’ muscle relaxants. One study published by Boggett et al (2020) was removed due to their method incorporating confounding variables that significantly decreased the reliability of the results: they used sugammadex with a deep neuromuscular block but neostigmine with a moderate block Two incomplete clinical trials were removed after contacting the authors, as both confirmed that the trials were still in the recruitment stage (CN-02674527 2024, CN-02704924 2024). Five studies were included in the review (Oh et al 2016, Pişkin et al 2016, Batistaki et al 2017, Muedra et al 2022, Rössler et al 2025).

### Comparison of studies included

The five studies identified for inclusion collectively reported interventions in 49,910 patients: 42,817 patients received neostigmine and 7093 patients received sugammadex. A summary of the study characteristics is shown in Table 1. Of the five studies, two were randomised, prospective, controlled, double-blind studies, (Batistaki et al 2017, Pişkin et al 2016) and three were retrospective cohort studies (Muedra et al 2022, Oh et al 2016, Rössler et al 2025). A mixture of diagnostic tools was used to detect delirium and POCD. Two studies (Batistaki et al 2017, Pişkin et al 2016) opted for the Mini-Mental State Examination (MMSE) with one additionally utilising the Montreal Cognitive Assessment (MoCA), two studies (Oh et al 2016, Rössler et al 2025) opted for the Confusion Assessment Method (CAM), brief Confusion Assessment Method (bCAM) or CAM-ICU, and one study (Muedra et al 2022) used the Postoperative Quality Recovery Scale (PQRS) (Martini et al 2015). Of the five studies, three studies (Batistaki et al 2017, Pişkin et al 2016, Rössler et al 2025) looked at patients undergoing any surgical procedure involving GA, one study (Oh et al 2016) looked at patients who had surgery for hip fractures, and one study (Muedra et al 2022) looked at patients undergoing aortic valve replacement procedure with an enhanced recovery after cardiac surgery (ERACS) approach. Different ages were used for the inclusion criteria, with two opting for > 60 years (Batistaki et al 2017, Muedra et al 2022), one > 65 years (Oh et al 2016), one > 45 years (Rössler et al 2025) and one > 18 years (Pişkin et al 2016). The characteristics of each study are summarised in Table 1. The outcomes and conclusions of each study are summarised by Table 2.

**Table 1:** Study characteristics

**Study** *(Author, Year)*	**Title**	**Study location**	**Aims**	***Study design***	**Study setting**	**Category of participants**	**Number of participants**	**Age** **±** **1 SD**	**Assessment tools**	**How was the assessment performed**	**Study funder/sponsor**	**Conflicts of interest**
Rössler et al (2025)	Association of Intravenous Neostigmine and Anticholinergics or Sugammadex with Postoperative Delirium: A Retrospective Cohort Study	Cleveland Clinic Main Campus United States	Association of intravenous neostigmine and sugammadex with postoperative delirium in clinical practice	*Observational cohort study*	Hospital	Inclusion: ASA I to III Exclusion: Delirium or dementia at baseline, history of delirium, simultaneous administration of neostigmine and sugammadex	49468 overall N 42,587 S 6881	N 57.6 ± 15.4 S 59.8 ± 15.3	bCAM or CAM-ICU	Postoperative delirium: 7 am on the first postoperative day 1 until day 4 or discharge; Early delirium: positive bCAM in first 24 h after extubation	Swiss National Science Foundation Early Postdoctoral Mobility Fellowship	None
Oh et al (2016)	Postoperative Delirium in Elderly Patients Undergoing Hip Fracture Surgery in the Sugammadex Era: A Retrospective Study	Konkuk University Medical Center South Korea	Evaluate the effects of sugammadex on POD in elderly patients who underwent hip fracture surgery	*Retrospective cohort study*	Hospital	Inclusion: Hip fracture patients in elderly Exclusion: Age < 60; preoperative neurological/psychological problem	174 overall N 96 S 78	N 75 ± 9 S 76 ± 7	CAM or CAM-ICU	Survey given if patient experienced signs of delirium confirmed by the surgeon. Psychiatrist would perform tool in postop patients.	National Research Foundation of Korea funded by the Ministry of Science, ICT and Future Planning	None
Batistaki et al (2017)	Effect of sugammadex versus neostigmine/atropine combination on postoperative cognitive dysfunction after elective surgery	Attikon University Hospital Greece	Effects of sugammadex and neostigmine/atropine on postoperative cognitive dysfunction (POCD) in adult patients after elective surgery	*Prospective, randomised, double-blind controlled trial*	Hospital	Inclusion: Age > 40; ASA I to III; Exclusion: Preoperative MMSE < 22; psychiatric disorders; allergies to protocol drugs; patient refusal	160 overall N 82 S 78	N 61.25 S 61.64	MMSE, clock drawing test and Isaacs Set test	POCD was defined as a decline equal to or more than one standard deviation in at least two of the three cognitive tests performed Measured before, 1 h postoperative and at discharge or 7 days postoperative if not discharged	Not reported	Not reported
Muedra et al (2022)	Potential Neuroprotective Role of Sugammadex: A Clinical Study on Cognitive Function Assessment in an Enhanced Recovery After Cardiac Surgery Approach and an Experimental Study	La Ribera University Hospital Spain	Understand if sugammadex could optimise the quality of postoperative cognitive function and overall recovery through a neuroprotective effect	*Single-centre, observational, and prospective pilot study*	Hospital	Inclusion: Age > 65; Exclusion: Patients with psychiatric/CNS disorders; allergies to protocol drugs	21 overall N 7 S 14	N 70 ± 5 S 74 ± 3	PQRS	Baseline using PQRS 12–24 h before surgery (T0) and then 30 min after extubation (T1), 24 h (T2), and 72 h (T3) after surgery, and finally 30 days after discharge (T4)	Merck Sharp & Dohme Spain and CEU Cardenal Herrera University.	None
Pişkin et al (2016)	The effect of sugammadex on postoperative cognitive function and recovery	Bülent Ecevit University Turkey	Understand the effect of sugammadex on postoperative cognitive function and recovery	*Randomised, prospective, controlled, double-blind study*	Hospital	Inclusion: Between 18 and 60 years of age; ASA I or II Exclusion criteria: Neuropsychiatric disease, GCS < 15, and postoperative MMSE score < 23 and MoCA score < 21	87 overall N 45 S 42	N 37.38 ± 11.95 S 32.07 ± 11.50	MMSE and MoCA	Patient required a Modified Aldrete Recovery Score ⩾ 9 prior to surgery. MMSE performed postoperatively then 1 h later the MoCA test	Not reported	None

**Table 2:** Study outcomes and conclusions

**Study ID** *(Author, Year)*	**General outcome**	**Specific outcome (POD and POCD incidence/recovery)**	**Odds ratio (POD and POCD incidence)**	**Author’s conclusion**	**Limitations of study as reported by authors**
Rössler et al (2025)	No significant difference in POD incidence between each intervention	POD incidence before propensity weighting: S 1.09% N 0.78% POD incidence after propensity weighting: S 1.09% N 0.82%	POD OR: 1.33 95% CI, 0.91–1.95 p = 0.147	Postoperative delirium was no less common with either intervention. Neostigmine and sugammadex might have a similar risk for delirium in adults having noncardiac surgery, and selection of neuromuscular block reversal agents probably does not help to prevent postoperative delirium.	Selection bias in more receiving neostigmine over sugammadex. Sugammadex might be preferred to mitigate pulmonary complications which may introduce bias as sugammadex might be given to sicker patients but ASD < 0.1 for ASA status. Low incidence of delirium despite large sample size. Central effects of atropine in association with postoperative delirium.
Oh et al (2016)	No significant difference in POD incidence between each intervention	POD incidence: S 33.3% [26/78] N 36.5% [35/96] p = 0.750	Not reported	Sugammadex did not reduce POD or pulmonary complications compared to conventional cholinesterase inhibitors, despite reducing time to extubation and postoperative hypoxia in elderly patients who underwent hip fracture surgery under general anaesthesia.	Effects of atropine. May have missed signs of hypoactive delirium as not all patients were screened by psychiatrists and selection was based on surgeon’s analysis. TOF ratio at extubation.
Batistaki et al (2017)	No significant difference in POCD incidence between each intervention	POCD incidence at 1 h postoperative: S 23 % N 28 % POCD incidence at discharge: S 5.4 % N 10 % N: p = 0.55 S: p = 0.27	Not reported	The incidence of POCD was similar in both groups at 1 h postoperatively and at discharge. There are no clinically important differences in the incidence of POCD after neostigmine or sugammadex administration	Baseline differences in types of surgery (higher prevalence of major surgery in the sugammadex group as well as a lower baseline CDT score). Would require larger study with testing that should extend for longer time periods, and ideally be stratified for surgical procedures and age group.
Muedra et al (2022)	Improvement shown in postoperative cognitive recovery by sugammadex group in patients undergoing aortic valve replacement procedure by the ERACS approach	POCD recovery (global domain): T1 (30 min): N 0/7 (0%), S 0/14 (0%) p = 0.127 T2 (24 h): N 3/7 (42.9%), S 3/14 (21.4%) p = 0.305 T3 (72 h): N 3/7 (42.9%), S 6/14 (42.9%) p = 1 T4 (30 d): N = 3/7 (42.9%), S = 12/14 (85.7%) p = 0.040	Not reported	Sugammadex was associated with favourable postoperative recovery in cognitive domains particularly 30 days after surgery in patients undergoing aortic valve replacement	Very small sample size. Higher doses of sugammadex not explored. Longer periods of study would have been better.
Pişkin et al (2016)	Although there was a reduction in MoCA and MMSE scores in both Group S and Group N between preoperative and postoperative scores, there was no statistically significant difference noted	MMSE: T0 (pre-op): S 26.98 ± 1.957, N 27.00 ± 2.286 p > 0.05 T1 (post-op): 26.90 ± 1.936, N 25.82 ± 2.724 p > 0.05 MoCA: T0 (pre-op): S 23.26 ± 1.988, N 23.56 ± 2.482 p < 0.05 T1 (post-op): S 23.17 ± 2.294, N 23.04 ± 2.868 p > 0.05	Not reported	Better cognitive performance could not be proved in the sugammadex compared to the neostigmine.	Limited in project duration so backup groups not included. Postoperative cognitive evaluation was completed only during the early period. Low doses of sugammadex used.

### Risk of bias

**Figure 3:**
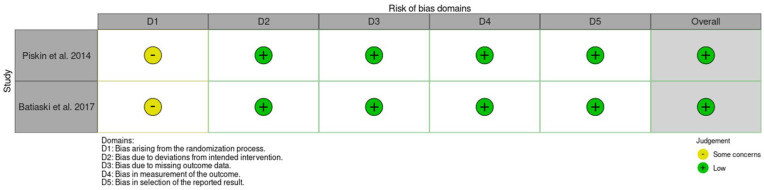
RoB-2 results summary table

**Figure 4:**
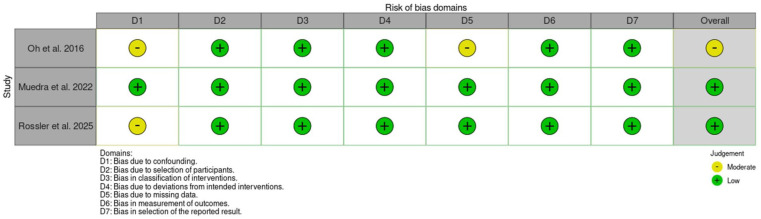
ROBINS-I results summary table

Two reviewers conducted independent assessments of the risk of bias utilising a revised Cochrane risk of bias tool. The RoB 2 tool (Sterne et al 2019) was employed to evaluate the risk of bias in randomised trials (Figure 3) while the ROBINS-I tool (Sterne et al 2016) was used for assessing the risk of bias in non-randomised studies of interventions (Figure 4). Overall, four studies (Batistaki et al 2017, Muedra et al 2022, Pişkin et al 2016, Rössler et al 2025) showed a low risk of bias while one (Oh et al 2016) raised some concerns.

### Effects on postoperative cognitive function

Four of the five studies showed neither statistical nor clinical differences in cognitive function between using sugammadex or neostigmine as a reversal agent, as summarised in Table 3 (Batistaki et al 2017, Oh et al 2016, Pişkin et al 2016, Rössler et al 2025). Only the observational study by Muedra et al (2022), who looked at patients undergoing cardiac surgery with the ERACS approach, noted favourable outcomes postoperatively, especially 30 days after surgery, when using sugammadex. Statistical significance is reported in the ‘global’ category, which assessed the patient’s perspective on their own ‘working capacity, daily activities, clarity of thought’ and ‘satisfaction with the anaesthetic care’ (42.9% for neostigmine, 85.7% for sugammadex with a p-value of 0.040). The word list category also showed statistical differences, with sugammadex patients able to remember more words from a previously read list than patients administered neostigmine (92.9% for sugammadex and 57.1 % for neostigmine, with a p-value of 0.049). However, the authors concluded that these statistically significant differences are unlikely to translate into clinical significance, as the differences are minor.

**Table 3:** Summary of overall results from each study

Study (year)	Incidence of delirium or POCD for patients receiving neostigmine	Incidence of delirium or POCD for patients receiving sugammadex	Recovery rates from anaesthesia back to cognitive baseline for patients receiving neostigmine	Recovery rates from anaesthesia back to cognitive baseline for patients receiving sugammadex
Rössler et al (2025)	0.82%	1.09 %	Not provided	Not provided
Oh et al (2016)	36.5%	33.3 %	Not provided	Not provided
Batistaki et al (2017)	At 1 H: 28 % At discharge: 10 %	At 1 H: 23 % At discharge: 5.4 %	Not provided	Not provided
Muedra et al (2022)	Not provided	Not provided	At 24 h: 42.9 % At 72 h: 42.9 % At 30 days: 42.9%	At 24 h: 21.4 % At 72 h: 42.9 % At 30 days: 85.7 %
Piskin et al (2016)	Not provided	Not provided	Not provided	Not provided

Instead of reporting the incidence for delirium or POCD, Pişkin et al (2016) listed numerical MMSE and MoCA scores preoperatively (T_0_) and postoperatively (T_1_), as shown in Table 4. The postoperative (T_1_) time point was defined as when the Modified Aldrete Recovery Score (MAS) was ⩾ 9, indicating that a patient can be discharged from the post-anaesthesia care unit (PACU) after surgery. This study revealed that the T_1_ mean was lower than the T_0_ mean in both groups, but there was no statistical significance between using neostigmine or sugammadex.

**Table 4:** MMSE and MOCA at times T_0_ and T_1_

	Sugammadex ± 1 SD	Neostigmine ± 1 SD
MMSE at T_0_	26.98 ± 1.957	27.00 ± 2.286
MMSE at T_1_	26.90 ± 1.936	25.82 ± 2.724
MoCA at T_0_	23.26 ± 1.988	23.56 ± 2.482
MoCA at T_1_	23.17 ± 2.294	23.04 ± 2.868

In the MMSE, scores of 25–30 out of 30 are considered normal, 21–24 as mild, 10–20 as moderate and below 10 as severe impairment. In the MoCA, scores of 26–30 out of 30 are considered normal, 18–25 as mild, 10–17 as moderate and below 10 as severe impairment.

## Discussion

This systematic review compared the effects of sugammadex and neostigmine on postoperative delirium and POCD. It was hypothesised that sugammadex would be beneficial due to a superior ability to reverse rocuronium and vecuronium and reduce the complications of a residual neuromuscular block. A systematic search resulted in five relevant papers (Batistaki et al 2017, Muedra et al 2022, Oh et al 2016, Pişkin et al 2016, Rössler et al 2025). Four papers concluded that there was no statistical difference in the incidence of POCD and delirium between the two reversal agents (Batistaki et al 2017, Oh et al 2016, Pişkin et al 2016, Rössler et al 2025). One paper showed some statistically significant improvements in certain domains when sugammadex was given, but the authors concluded that the results were unlikely to be clinically significant (Muedra et al 2022).

POCD and delirium have multifactorial causes. While theoretically the two drugs may show some difference in cognitive effects on patients, the effect is likely insignificant compared to other patient, anaesthetic and surgical factors (Krause et al 2020). Only Muedra et al (2022) reported a statistical difference, with lower incidences of POCD with sugammadex on patients undergoing cardiac surgery with the ERACS approach. As this study controlled for the type of surgery and recovery protocol, and thus more likely to reduce confounding patient, surgical and anaesthetic factors, this study’s methodology could have made the potential effects of the different neuromuscular reversal agents more pronounced.

Rössler et al (2025) shows a much lower incidence of delirium and POCD in all patients than the other reports. This could be because cardiac surgery patients were excluded from this review, which has been shown to increase delirium and POCD. They did not control for the surgical procedure other than this, so other anaesthetic and surgical factors could have more of an effect on delirium and POCD than the choice of neuromuscular reversal agent, masking the difference between the drugs.

Four of the five studies demonstrated a low risk of bias (Batistaki et al 2017, Muedra et al 2022, Pişkin et al 2016, Rössler et al 2025). Only the study published by Oh et al (2016) exhibits moderate concern regarding the risk of bias, primarily due to uncertainties surrounding how patients were evaluated post-surgery. Oh et al (2016) determined if the patient was eligible for the POCD assessment through the surgeon identifying symptoms of delirium in the patient. Only patients identified as having delirium were assessed by a psychiatrist. However, especially in cases of hypoactive delirium, symptoms of delirium can be easily missed, leading to bias due to human factors and judgement.

While the report by Muedra et al (2022) had a low risk of bias across all domains, it is worth noting that the overall sample size was limited to 21 patients which limits the strength of statistical analysis. This is because this study used a logically robust methodology to stratify patients to minimise risk of bias. Through doing this, only a small population was included in the study. The PQRS assessment tool (Martini et al 2015) utilised in the study is also more subjective than other screening methods, which might increase the risk of bias. However, as the PQRS (Martini et al 2015) assessment is a widely accepted tool, the reviewers assessed the risk of bias to be minimal.

The study conducted by Rössler et al (2025) has a risk of bias in the D1 category due to unclear confounding variables. It is ambiguous regarding the doses of reversal agents administered and whether only GA was used, leading to questions over whether they could be directly compared. Furthermore, as it is a retrospective study, there are missing variables; for instance, the nutritional status or cancer history of the patients. Fewer patients received sugammadex, and it is acknowledged that cardiac patients especially were given sugammadex as it has been proven to have protective effects for this cohort of patients in comparison to neostigmine. However, patients undergoing cardiac surgery might have a different baseline from those receiving other surgery types, for instance length of surgery or comorbidities. Cardiac procedures have higher rates of POCD and delirium, which could affect the results (Wang & Wang 2024).

For the RCTs, both studies indicate bias stemming from the randomisation process. Pişkin et al (2016) stated they required 128 patients for statistical power, but a limited timeframe for data retrieval meant that backup groups were not included. A total of 128 patients were recruited and 41 patients dropped out, leaving only 87 people, limiting the strength of the evidence produced.

Batistaki et al (2017) acknowledged baseline differences in the surgical procedures performed, with a higher prevalence of major surgeries for the sugammadex patients. This could influence the cognitive function of the patients disproportionately. The research group acknowledged that a more extensive study should be undertaken and stratified for surgical procedures and age groups.

This review has several limitations. The studies use a range of modalities to assess cognitive function, making direct comparison between them difficult, and a meta-analysis in this topic not currently possible. Alongside the confounding factors of other drugs administered in the anaesthesia regime, this makes it harder to specifically compare the influence of neostigmine and sugammadex alone on delirium and POCD. Furthermore, only five reports were suitable for inclusion, raising concerns about the limited scope and potential gaps in reliability due to the lack of broader perspectives.

In conclusion, both sugammadex and neostigmine have a similar effect on the incidence of POCD and delirium. While sugammadex displays marginal improvement over neostigmine, this was found not to be clinically significant. This suggests that the choice of the neuromuscular reversal agent can be based on other factors such as anaesthetist preference, clinical suitability, expense or availability, rather than their effect on cognition. A further systematic review that includes the results of the two ongoing clinical studies currently collecting data should be performed at a later date to confirm these results.

## Appendix 1

**Table:** Full search terms used:

(MEDLINER ALL <1946 to October 17, 2024>	
#1	Sugammadex.mp. OR Sugammadex
#2	Neostigmine.mp. OR Neostigmine
#3	Emergence Delirium/ OR Delirium/ OR Postoperative Complications/ OR postoperative delirium.mp.
#4	Postoperative Cognitive Complications/ OR Cognitive Dysfunction/ OR Cognition/ OR Cognition Disorders/ or postoperative cognitive function.mp. OR Postoperative Complications/
#5	#3 OR #4
#6	#2 AND #5
#7	#1 AND #5
Embase <1974 to 2024 October 17>	
#1	Sugammadex.mp. or sugammadex/
#2	Neostigmine methyl sulphate/ OR neostigmine.mp. OR neostigmine/ OR glycopyrronium plus neostigmine methyl sulphate/
#3	Postoperative delirium.mp. or delirium/ or postoperative delirium/ or cognitive defect/ or postoperative complication/
#4	Cognition/ or postoperative cognitive function.mp. or postoperative cognitive dysfunction/
#5	#3 OR #4
#6	#2 AND #5
#7	#1 AND #5
#8	limit #7 to ("remove medline records" and yr="2008 -Current")
#9	limit #6 to ("remove medline records" and yr="2008 -Current")
Web of Science	
#1	ALL=(sugammadex)) OR ALL=(bridion)
#2	ALL=(Delirium)
#3	(((ALL=(Cognition)) OR ALL= (Cognitive)) OR ALL=(Memory)) OR ALL=(Mental)
#4	#2 OR #3
#5	#1 AND #4
#6	((ALL=(neostigmine)) OR ALL=(prostigmin)) OR ALL=(bloxiverz)
#7	#6 AND #4
CINAHL:	
#1	(MH “Sugammadex”) OR “sugammadex OR org 25969 OR selective relaxant binding agent”
#2	(MH “Neostigmine”) OR “neostigmine OR prostigmine OR prostigmin” OR “bloxiverz”
#3	“(postoperative delirium or post-operative delirium) OR (postoperative cognitive dysfunction or pocd)" OR (MH “Cognition”) OR (MH “Postoperative Complications”) OR (MH “Delirium”) OR (MH “Delirium, Dementia, Amnestic, Cognitive Disorders”)
#4	#2 AND #3
#5	#1 AND #3
COCHRANE library	
#1	Sugammadex OR Bridion OR Org25969 OR Selective relaxant Binding Agent
#2	Neostigmine OR Prostigmine OR Prostigmin OR Bloxiverz
#3	Delirium OR Cognition OR Cognitive function OR Memory
#4	Postoperative
#5	#3 AND #4
#6	#1 AND #5
#77	#2 AND #5
ClinicalTrials.gov	
#1	Postoperative Complications | Other terms: Cognitive Decline OR Delirium OR Cognitive Dysfunction | Neostigmine OR Prostigmin OR neostigmin OR bloxiverz
#2	Postoperative Complications | Other terms: Cognitive Decline OR Delirium OR Cognitive Dysfunction | selective relaxant binding agent OR org 25969 or sugammadex OR bridion
Google Scholar First 200 references included, sorted by most relevant from 2008-current	
#1	Search terms any of neostigmine or prostigmine or prostigmin or bloxiverz and cognition or delirium and postoperative
#2	Search terms any of sugammadex or org25969 or selective relaxant binding agent or bridion and cognition or delirium and postoperative
FDA	
#1	“Neostigmine”
#2	“Prostigmin”
#3	“Prostigmine”
#5	“Bloxiverz”
#6	“Sugammadex”
#7	“Bridion”
#8	“Selective relaxant binding agent”
#9	“Org 25969”
EMA	
#1	“Neostigmine”
#2	“Prostigmin”
#3	“Prostigmine”
#4	“Bloxiverz”
#5	“Sugammadex”
#6	“Bridion”
#7	“Selective relaxant binding agent”
#8	“Org 25969”
